# Chitosan nanoparticles as next-generation carriers for veterinary DNA vaccines: Mechanisms, immune responses, and translational prospects

**DOI:** 10.14202/vetworld.2025.3826-3838

**Published:** 2025-12-13

**Authors:** Miguel González-Lozano, José Alberto Cano-Buendía

**Affiliations:** 1 Facultad de Medicina Veterinaria y Zootecnia; Center for Teaching, Research and Extension in Swine Production, Universidad Nacional Autónoma de Mexico (UNAM), Cuidad Universitaria, 04510; Mexico City, Mexico; 2 Facultad de Medicina Veterinaria y Zootecnia; Department of Microbiology and Immunology, Universidad Nacional Autónoma de Mexico (UNAM), Cuidad Universitaria, 04510; Mexico City, Mexico

**Keywords:** chitosan nanoparticles, genetic immunization, mucosal vaccination, nanocarriers, nanotechnology in animals, One Health, plasmid DNA, vaccine delivery systems, veterinary DNA vaccines, viral diseases

## Abstract

Chitosan-based DNA nanoparticles have emerged as a promising next-generation platform for veterinary vaccines, addressing several limitations of conventional attenuated, inactivated, and recombinant formulations. Chitosan is a biodegradable, biocompatible, and low toxicity polymer with mucoadhesive properties that enhance cellular uptake and protect nucleic acids from enzymatic degradation. These characteristics make it an attractive candidate for delivering plasmid DNA encoding viral antigens across diverse animal species. Recent advances demonstrate that chitosan–DNA nanoparticles can induce robust humoral and cellular immune responses, stimulate mucosal immunity, and achieve high levels of protection in terrestrial livestock, poultry, fish, and crustaceans. A wide range of viral pathogens has been targeted using this approach, including Foot-and-Mouth disease virus, Newcastle disease virus, infectious bronchitis virus, spring viremia of carp virus, white spot syndrome virus, and infectious pancreatic necrosis virus. Depending on the species and formulation strategy, nanoparticles have been successfully administered intranasally, intramuscularly, intraperitoneally, or orally, highlighting their versatility for mass vaccination in both terrestrial and aquatic systems. Reported protection rates range from 60% to 100% in mammalian and avian models, while oral nanoparticle vaccines in shrimp and fish have demonstrated sustained immune activation and survival benefits. The ability to incorporate genetic adjuvants, such as cytosine-phosphate-guanine motifs, cytokines, or complement fragments, further enhances the immunogenicity of these platforms. Despite these promising results, several challenges remain. Most studies use small laboratory animals or controlled experimental settings, and data from large-scale field trials in cattle, pigs, and equines remain scarce. The stability of nanoparticle formulations during long-term storage, the scalability of manufacturing processes, and the standardization of dosing regimens require further investigation. Overall, chitosan–DNA nanoparticles represent a safe, flexible, and rapidly adaptable vaccine carrier system with significant potential to transform veterinary immunization. Their capacity to elicit mucosal and systemic immunity, enable needle-free delivery, and support DIVA-compatible vaccine design positions them as a valuable tool for controlling emerging and re-emerging viral diseases in the context of One Health.

## INTRODUCTION

The Food and Drug Administration (FDA) classifies chitosan as Generally Recognized As Safe (GRAS) [1] due to its biocompatibility, biodegradability, and low toxicity. As the global population increases, both humans and animals experience greater exposure to viral pathogens, intensifying the health and economic burden of viral diseases worldwide. This growing challenge has shifted modern medicine toward prevention rather than treatment. Although antiviral drugs are available, their long-term success is limited by mechanisms such as drug resistance, genetic drift, and genetic shift.

Vaccination remains the most effective preventive strategy; however, veterinary vaccines must be affordable, rapidly adaptable to circulating viral strains, and compatible with mass delivery through aerosol, oral,

or parenteral routes. Conventional first-generation vaccines (attenuated or inactivated) and second-generation platforms (recombinant proteins or recombinant vectors) have been widely used. In contrast, third-generation vaccines rely on plasmid or linear DNA/RNA that encodes a target antigen. Once the genetic material enters host cells, the antigen is expressed, folded, and post-translationally modified in vivo, eliminating the need for large-scale pathogen culture or complex protein purification processes [2]. The expressed protein is then recognized by the immune system, triggering humoral or cellular immunity depending on the adjuvant used. Several delivery technologies, such as gene guns, electroporation, and dermal patches, have been developed to enhance nucleic acid uptake [3]. Advances in nanotechnology now provide additional strategies to improve cellular internalization and gene transfer efficiency, allowing DNA vaccines to induce stronger protective immune responses and reduce morbidity and mortality associated with viral infections.

To further enhance the delivery of genetic vaccines, multiple methodologies are being evaluated to improve transfection efficiency and support large-scale, pharmaceutical-grade production. One promising approach involves formulating DNA within chitosan nanoparticles, which protect nucleic acids from endonuclease degradation and acidic environments. Their mucoadhesive properties promote interaction with mucosal surfaces, facilitating targeted uptake by specific tissues [4]. Chitosan–DNA nanoparticles have therefore emerged as a versatile platform for delivering genetic material in applications ranging from antibacterial, antiviral, and antiparasitic vaccines to gene therapy and cancer treatment. Their suitability stems from chitosan’s inherent biocompatibility, biodegradability, and minimal immunogenicity [5].

Veterinary vaccine development poses unique challenges because aquatic, avian, and mammalian species exhibit diverse immune responses, dosing requirements, and delivery constraints. Nonetheless, several studies have shown that chitosan enhances immune responses, such as improving protection against avian influenza, highlighting its potential as an efficient carrier for veterinary genetic vaccines [5, 6].

Although numerous studies have demonstrated the potential of chitosan-based DNA nanoparticles for delivering genetic vaccines across different animal species, the available evidence is highly fragmented. Existing reports vary widely in nanoparticle formulation methods, antigen design, dosing regimens, animal models, and immunization routes, making it difficult to identify standardized parameters for effective vaccine development. Moreover, most investigations remain confined to laboratory-scale trials in small animals, with limited data on large livestock species, field applicability, long-term stability, manufacturing scalability, and regulatory pathways. There is currently no comprehensive review that integrates these scattered findings and critically evaluates the translational challenges, species-specific considerations, and comparative outcomes of chitosan–DNA–nanoparticle vaccines in veterinary medicine.

The aim of this review is to consolidate and critically analyze current evidence on the use of chitosan-based DNA nanoparticles as vaccine carriers for viral diseases in animals. Specifically, the review seeks to (i) summarize nanoparticle formulation strategies and their influence on gene delivery efficiency, (ii) compare immunogenic and protective outcomes across terrestrial and aquatic species, (iii) highlight advantages and limitations of chitosan nanocarriers for veterinary vaccination, and (iv) identify the key scientific, technological, and regulatory barriers that must be addressed to advance these platforms toward large-scale, field-ready veterinary vaccines.

## CHARACTERISTICS OF CHITOSAN

### Origin and Chemical Structure

**Figure 1:**
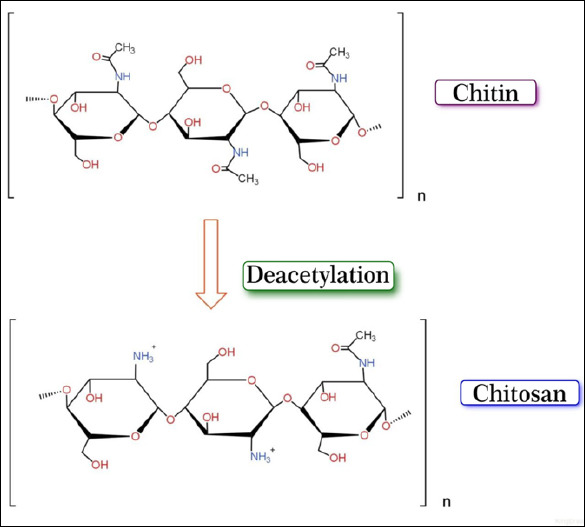
Chemical structures of chitin and chitosan. Two acetyl groups are removed by deacetylation, generating a positively charged chitosan.

Chitosan is a naturally occurring polysaccharide derived from the deacetylation of chitin (Figure 1), a structural component found in the exoskeletons of crustaceans (shrimp and crab), insects, and fungal cell walls [7]. It is the second most abundant nitrogen-containing polysaccharide after cellulose and is composed of repeating N-acetyl-glucosamine and D-glucosamine units linked through β-(1→4) glycosidic bonds [8]. Deacetylation, achieved through chemical or biological processes, introduces free amino groups that define the physicochemical and biological behavior of chitosan [9].

### Charge Properties and DNA Binding

Chitosan carries a positive charge, while DNA is negatively charged, enabling spontaneous electrostatic interactions that form nanoscale complexes known as nanoplexes or polyplexes. The stability and transfection efficiency of these complexes depend on factors such as molecular weight, degree of deacetylation, nitrogen/phosphate (N/P) ratio, ionic strength, additives, formulation method, and the intended administration route [10].

### Biodegradability and Cellular Interactions

**Figure 2:**
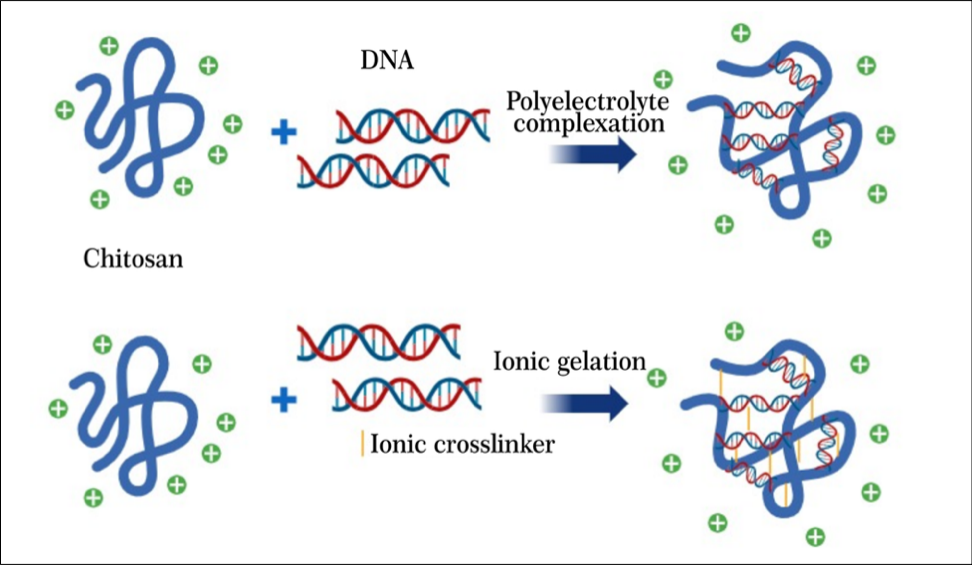
Main fabrication methods for DNA nanoparticles. A polyelectrolyte complexation, where DNA and chitosan interactions are made by charges; B ionic gelation, where TPP can function as a crosslinker.

The biodegradability of chitosan stems from its glycosidic bonds, which can be enzymatically cleaved by proteases, lysozymes, and chitosanases. Degradation yields non-toxic oligosaccharides, while the polymer’s cationic nature enhances its interaction with negatively charged cell membranes, promoting uptake [11]. Several preparation methods, such as ionic gelation, cross-linking, and covalent cross-linking, are used to generate chitosan–DNA nanoparticles (Figure 2). Although these approaches allow rapid nanoparticle production, they may influence immune activation and transfection efficiency [12].

### Nanoparticle Behavior and Biological Distribution

Nanoparticle clearance is strongly size-dependent. Larger particles tend to become trapped in small capillaries, such as those in the lungs, whereas nanoparticles smaller than 200 nm exhibit longer circulation times and preferentially accumulate in organs, such as the liver and spleen [13]. Chitosan also demonstrates mucosal adhesion via electrostatic interactions with mucins, facilitating uptake by M cells and supporting oral or mucosal administration. Additionally, its proton-absorbing capacity contributes to mild analgesic effects during inflammatory processes [14].

### Influence of Molecular Weight (MW)

Molecular weight significantly affects chitosan’s bioactivity and functional performance. Low-molecular-weight chitosan (<20 kDa) exhibits higher bioactivity than high-molecular-weight variants (>120 kDa). When MW exceeds 30 kDa, protonation of amino groups using acids such as acetic, hydrochloric, citric, lactic, or pyruvic acid is required to solubilize the polymer in aqueous environments [8, 15]. MW also influences nanoparticle size, stability, cellular targeting, and transfection efficiency, making it a critical parameter in nanoparticle formulation [16].

## NANOPARTICLE FORMULATION

### Definition and Preferred Size Range

Nanoparticles (NPs) are typically defined as particles ranging from 10 to 1000 nm in diameter [17]. In practice, sizes below 100 nm are often preferred because they exhibit unique physicochemical properties, including a high surface-area-to-volume ratio and increased reactivity. These attributes enhance their ability to interact with biological membranes and efficiently deliver genetic material into target cells.

### Classification of Nanoparticles

Nanoparticles are broadly categorized as organic or inorganic [11, 17, 18]. This review focuses on organic nanoparticles, particularly those based on chitosan. Organic NPs are sensitive to environmental conditions such as temperature and electromagnetic radiation, yet they offer significant advantages, including biodegradability, low toxicity, biocompatibility, and controlled release of encapsulated compounds [19].

### Chitosan Nanoparticles as Gene Delivery Systems

Chitosan nanoparticles possess several desirable nanostructural features: small size, large surface area, and a positively charged surface. These characteristics promote self-assembly with negatively charged DNA, forming stable nanoplexes that are often more efficient and stable than lipoplex-based delivery systems. Chitosan NPs enter cells primarily through electrostatic interactions or passive endocytosis, protecting DNA from nuclease degradation and preventing non-specific dispersion—limitations commonly associated with naked DNA [8]. This makes them highly promising for non-viral gene delivery applications.

### Biodistribution and Biological Barriers

The distribution of nanoparticles in vivo is strongly influenced by the route of administration. Intravenously administered NPs must navigate plasma proteins, vascular endothelium, and interstitial tissues before reaching target cells. They are internalized via adsorption and endocytosis, after which the payload is released into the cytoplasm. Additional barriers, such as the blood–brain barrier, present tightly regulated endothelial junctions that restrict nanoparticle passage. Along their journey, nanoparticles may interact with enzymes, proteoglycans, serum proteins, and extracellular matrix components, leading to aggregation and clearance by the mononuclear phagocytic system [20].

### Nitrogen/Phosphate (N/P) Ratio

N/P ratio refers to the balance between positively charged amino groups in chitosan (N) and negatively charged phosphate groups in DNA (P). This ratio is crucial for determining nanoparticle characteristics, such as net surface charge, complex stability, and transfection efficiency.

- Low N/P ratios produce unstable, inefficient complexes with reduced ability to protect DNA and facilitate cellular uptake.
- High N/P ratios improve complex formation and intracellular delivery but may increase cytotoxicity due to excess cationic polymer accumulation [21]

An optimal N/P ratio ensures efficient nucleic acid encapsulation with the minimal amount of polymer required to maintain safety and functionality. However, excessively high ratios can lead to overly stable complexes, non-specific aggregation, and unintended interactions with cellular or vascular components. Chitosan also influences immune cell activity, particularly in granulocytes and macrophages, which play important roles in antigen presentation [9]. These immunomodulatory properties support its use in nanoparticle-based vaccine systems.

## DNA–NANOPARTICLE VACCINE APPLICATIONS

The following section outlines various strategies used against different viral diseases, detailing the corresponding animal models, doses, and administration routes. All summarized data are presented in Table 1.

**Table 1:** DNA-nanoparticle vaccines against different viral diseases

ID	NP size (nm)	Administration route	Protection rate	Preparation method	Animal model	Dose	Immunization frequency
A	462.5	IN	60	Emulsion-diffusion-evaporation	Guinea pig and rat	200 µg	Guinea pigs: 3 times every 2 weeks; Rats: 2 times every 2 weeks
B	130–500	IN or IM	60–71.8	Complex coacervation	Guinea pig	20 µg	Once
C	500	IN	75	Emulsion-diffusion-evaporation	Cattle	1–1.3 mg	3 times (days 1, 4 and 7)
D	400–500	IN	100	Emulsion-diffusion-evaporation	Guinea pig	200 µg	Twice every 2 weeks
E	199.5	IN	100	Complex coacervation	SPF chicken	200 µg	Once
F	309.7	IN	100	Polyelectrolyte complex	Chicken	200 µg	Twice every 2 weeks
G	202.3	IN	100	Polyelectrolyte complex	Chicken	200 µg	Once
H	95	IN	ND	Complex coacervation	SPF chicken	100 or 2×200 µg	Once or twice
I	95	IN	ND	Complex coacervation	Chicken	100 µg	Twice every 14 days (boost with protein)
J	70.67	IM	ND	Complex coacervation	SPF chicken	100 µg	3 times every 2 weeks
K	140.9	IM	90	Complex coacervation	SPF chicken	150 µg	Twice every 14 days
L	204.43	IP	63.33 after 3 dpv; 85.33 after 21 dpv; 59.33 after 70 dpv	Complex coacervation	Zebrafish	2 µg/g	Once
M	287.62	IP	83.78 after 28 dpv; 72.97 after 70 dpv	Double-emulsion	Common carp	2 µg/g	Once
N	ND	Oral	90 after 7 dpv; 76.66 after 14 dpv; 56.66 after 30 dpv	Complex coacervation	Shrimp (*Penaeus monodon*)	1 mg/10 g feed paste	7 days / twice a day
O	30–60	Oral	ND	Ionic gelation	Shrimp (*Penaeus monodon*)	100 µg/10 g	Once
P	161.56	IM	100% after 30 dpv; 85% after 15 dpv; 15% after 3 dpv	Complex coacervation	Dehua black chicken	1 µg/g body weight	Twice every 14 days
Q	150	Oral/nasal	ND	Ionic gel	Chicken	ND	Three times every 14 days
R	ND	Oral	70 with 10 µg; 85 with 25 µg	Ionic gelation	Rainbow trout	10 and 25 µg/fish	Twice every 14 days
S	100–200	Oral	54	Ionic gelation	Grass carp	30 mg/kg feed	Days 1, 3, 5, 15, 17, 19

A–D = Foot-and-mouth disease, E–G = Newcastle disease, H–K = Infectious bronchitis virus infection, L = Spring viremia of carp virus infection, M = Spring viremia of carp virus infection (SVCF strain), N–O = White spot syndrome virus infection, P = Avian reticuloendotheliosis, Q = Avian influenza virus H9N2 infection, R = Infectious pancreatic necrosis virus infection, S = Grass carp hemorrhagic disease, IN = Intranasal, IM = Intramuscular, IP = Intraperitoneal, ND = Not determined, dpv = Days post-vaccination, SPF = Specific-pathogen-free

### Foot-and-Mouth Disease

Foot-and-Mouth Disease (FMD) is a severe, highly contagious viral disease that negatively impacts the livestock industry due to its high morbidity. The presence of cloven-hoofed animals must be reported to the Office International des Epizooties. Currently, commercial vaccines are based on inactivated pathogens, which require the virus to be grown for its preparation, in addition to the fact that there is a great antigenic diversity and they do not induce long-term immunity [22].

In one study, P12A (polypeptide capsid) was evaluated with 3C protease, along with the *IL-6* gene, either before or between the viral genes. NPs were prepared with poly(lactide-co-glycolide) (PLGA) and chitosan using the emulsion-diffusion-evaporation technique. Chitosan was used because of its ability to penetrate mucosal surfaces, and NPs were administered intranasally. Guinea pigs were immunized with a genetic construct, and two types of cassettes were used. In one version, the *IL-6* gene was between the evaluated genes, and the immunized group showed higher immunoglobulin (Ig)G and IgA titers. When the *IL6* gene was present before the virus genes, a greater cellular response was induced; however, when IL6 was present at the beginning of the cassette, protection was 60% and only 20% in the other construction [23].

In a study by Nanda *et al*. [24], an immunization was performed pigs were immunized with a construct encoding the *VP1* gene and the full-length outer membrane protein A (OmpA) of *S. typhimurium* to recruit dendritic cells. NPs were 80–500 nm in size, and when guinea pigs were immunized with 20 µg of DNA, neutralizing antibody titers were up to 1.8 Log10 after 30 days post-immunization (dpi), as well as a greater titer of IgG2, related to a Th1 response, necessary for viral infection control. However, its performance was similar to the inactivated vaccine group.

In a similar study, two types of NPs were prepared: chitosan–DNA and chitosan–inactivated FMD virus NPs, generated using the emulsion-diffusion-evaporation method [25]. Both NPs were administered 150 mg IN, and the immune response was analyzed. A higher sIgA level was detected 10 days post-vaccination (dpv), and no IgG high titers were induced in vaccinated animals. This type of vaccination provided 75% protection in cattle, which is explained by the presence of sIgA, which blocks infection of respiratory epithelial cells and could also help reduce viral shedding by reducing viral transmission. The DNA-based NPs protected cattle better than the NPs that included an inactivated virus, making it a promising candidate for vaccine development.

The polypeptide P1-2A (a precursor of a structural protein) is reportedly cleaved by protease 3C, making it a vaccine candidate. These genes were absorbed into amino-functionalized mesopores silica NPs [26] and used with another plasmid encoding cytosine-phosphate-guanine oligodeoxy-nucleotides as an adjuvant. In some variants, the porcine IFN-α was also included. The obtained NPs also contained PLGA and were administered to guinea pigs IN with NPs containing 200 µg of the plasmid DNA. The FMD virus can infect cattle, pigs, and sheep, so mucosal protection is important. Interferon is well known for inducing the expression of antiviral proteins and increasing the activity of natural killer cells, which is why it was included in some experimental groups. In this study, silica NPs were shown to protect the plasmid from degradation by DNAse I and to remain stable. They could also induce higher titers of neutralizing antibodies than the naked plasmid. It is well known that the mucosal immune system is connected. However, sIgA was detected in the lungs but not in the saliva or feces. Immunization showed a higher stimulation index (lymphocyte proliferation assay), which could be due to the inclusion of the *IFN-α* gene as well as the CpG sequence.

All FMD reports used nasal NP delivery, achieving protection rates of 60% to 100%. These protection studies were performed in guinea pigs that generated humoral immune responses, using the structural protein *VP1* and the viral protease 3C. In most of the studies, adjuvants were used [23, 25, 26], where guinea pigs were used and 100% of protection was reported [26]. However, when cows were evaluated, no adjuvant was used and the amount of DNA for vaccination (1–1.3 mg) was the highest, reaching 75% of protection [25]. Most studies were performed in guinea pigs; however, FMD is more closely related to cattle, which require higher doses and the evaluation of adjuvants. Another important aspect is that the delivery route was nasal, which could facilitate the vaccination process of herds.

### Newcastle Disease Virus

The Newcastle virus (NCV) is distributed worldwide and affects birds, causing nervous, respiratory, enteric, and reproductive problems. There are commercial vaccines against this virus, which are attenuated or recombinant vaccines, which require biocontainment that must be considered during vaccine production, as well as cold chain. In attenuated vaccines, the risk of reversion always exists.

When gene vaccines are administered intramuscularly (IM), the DNA must transfect antigen-presenting cells, which results in weak immune responses and the need to increase the amount of DNA per immunization.

Zhao *et al*. [27] reported the generation of nanoparticles by complex coacervation containing the NDV *F* gene. NPs were administered IN and IM, and the results were compared with empty NPs and naked plasmids. Chickens were immunized with 200 µg of plasmid, and IgG and IgA titers were induced. Chickens were challenged with the highly virulent F48E9 strain. The mean size of the generated nanoparticles was 199.5 nm. IgG and IgA titers were higher than those of the naked plasmid immunized group, and the highest titers were observed when IN immunization was performed. In relation to the percentage of protection, IN and IM immunized birds obtained 100% and 80% protection, respectively, whereas the naked DNA group generated 60% protection.

In a previous study by Zhao *et al*. [28], NPs were synthesized using the polyelectrolyte complex method in which the C3d6 molecule, which is a complement protein, was added to the plasmid and used as an adjuvant. Chickens were immunized and challenged as previously reported, and the IgG and IgA titers of the immunized birds were higher when IN administration was used. IL-2, IL-4, and IFN-γ were also elevated, which are associated with the Th1 response. Challenges caused 100% protection when NPs were administered in the IN and 71% for the IM route, and the naked plasmid generated 43% of protection. A variant of this study used N-2-Hydroxypropyl trimethyl ammonium chloride chitosan (N-2-HACC) as an adjuvant and obtained similar results [29].

Poultry is a dynamic field in food production where nanoparticle vaccines were evaluated using intranasal delivery and the dose was consistent in all studies (200 µg/bird). It is important to note that the *F* gene was targeted by the immune response. Studies using adjuvant (C3d6) and without adjuvant protection rates were 100%. It could be interesting to evaluate the duration of the immune response with and without an adjuvant. Another important remark is that full protection was detected with a single immunization dose and the intranasal route, which is commonly used in poultry.

### Infectious Bronchitis Virus Infection

The infectious bronchitis virus (IBV) belongs to the Coronaviridae family and causes respiratory problems in chickens, including sneezing, lethargy, and difficulty breathing. Consequently, there is little weight gain and a low carcass score. The health status of birds can be complicated by opportunistic infections. Commercially available attenuated vaccines are used to protect birds; however, antigenic variability in the spike glycoprotein (S) generates different serotypes that escape from the immune response. Attenuated vaccines allow vaccine viruses to recombine with circulating viruses; these viral strategies require the development of protective vaccines.

In a study by Chandrasekar *et al*. [30], a vaccine that codes for N protein, as NPs, was conjugated with Quail-A adjuvant. NPs with a size ≤100 nm were administered IN at 100 µg/immunization. Vaccines were evaluated in chicken embryos, and the weight gain of hatched chicks was evaluated. Birth rates and weight gain were not affected. IgA in tears was evaluated, and NPs with Quail-A induced higher immunoglobulin titers than the non-adjuvant NP group, with no difference from the group immunized with naked plasmid. Proliferation assays showed that the NP-adjuvant group showed a higher rate of stimulation of CD8+ and TCRγδ+ T lymphocytes. Moreover, the NP with adjuvant group showed a lower viral load and consequently reduced tracheal viral shedding. Immunized chickens also showed fewer clinical signs after the challenge.

In another study, the vaccination scheme was modified: vaccination began with DNA priming, followed by vector boosting with Modified Vaccinia Ankara (MAV). NPs and viral vector codification for N protein and IN vaccinations were performed using 100 µg of DNA [31]. The vaccination protocol was compared with another one in which two immunizations with the MAV were performed. IgY and IgA titers were quantified, and low titers were found in the serum and tears of chickens immunized by both schemes. A long-term T cell proliferation assay was performed, in which the heterologous immunized group (NPs-MAV) showed greater CD8+ and TCRγδ T lymphocyte proliferation. These results are consistent with the clinical evaluations and viral load- in tears and the trachea: chickens vaccinated with the heterologous scheme showed low values, whereas the homologous group did not show high values, demonstrating induction of the protective immune system. In the same trial, the adjuvant MPLA, a TLR4 ligand that stimulates the expression of pro-inflammatory genes, was included without altering results, indicating that the NP-viral vector-induced immune response was protective and robust.

Another study evaluated the effect of a DNA vaccine containing the S1 sequence of the M41 and CR88 strains, making it a bivalent vaccine [32]. Saponin was included as a component in the nanoparticles, and they were administered IM three times with 100 µg each. The immunized birds produced anti-IBV antibodies, and antibody levels increased after the second boost. CD4+ and CD8+ lymphocyte counts were higher in chickens immunized with NPs as well as with the bivalent construct. Both groups showed the lowest virus shedding compared with the other groups. This vaccine has shown potential for development against IBV.

In another study, a cassette containing 4 domains of neutralizing epitopes and 4 T cell epitope sequences of the *S* gene was constructed [33]. The plasmid included a CpG motif. The NPs were 140 nm in size and were for IM immunization of specific pathogen-free chickens with 150 µg of plasmid DNA on days 0 and 14. Neutralizing antibody titers were higher than those with phosphate-buffered saline (PBS)or NPs containing the empty vector immunized groups, but there was no statistical difference. Enzyme-linked immunosorbent spot was performed to evaluate cellular immune response, and splenocytes of immunized chickens with NPs produced more IFN-γ and CD8+ and CD4+ T lymphocytes recognized the N261-280 peptide. In the protection trial, the NP vaccine provided 90% protection, whereas the groups immunized with PBS or with NPs containing an empty vector showed 60% and 40% mortality, respectively, which corresponded to clinical signs. Regarding viral load after the challenge, there was no significant difference among the three groups; however, NPs containing key epitopes elicited protective immune responses.

Coronaviruses are an important virus family that frequently change antigenic determinants, especially in the S protein. Here, we included different studies where adjuvants were used or not and when they were included in the formulations, the cellular immune response was induced. However, in all studies, at least 2 doses were evaluated, and the NP dose range was 150–200 µg. Unfortunately, only Quin *et al*. [33] reported 90% protection, and the remaining studies did not include this data. Additionally, this study used genes *S* and *N* to elicit the immune response, whereas the others used the genes alone. Studies included in this manuscript used IN and IM delivery routes. IN vaccination can be performed quickly; however, IM requires some experience for its application.

### Spring Viremia of Carp Virus

The spring viremia of carp virus causes a disease in cyprinid fish worldwide, resulting in economic losses. In one study, erythrocyte membranes were used as innate carriers of gene vaccines with mannose and chitosan [34]. NPs were prepared using plasmids encoding the SVCV *G* gene. After that, NPs were coated with erythrocyte membranes because they have a long circulation time, are stable, and are biocompatible. The obtained NPs had a size of 204 nm, and the zebrafish were immunized with 2 µg/g of weight and challenged at 3, 21, and 70 dpv. Nanoparticles were taken up by macrophages and demonstrated *in vivo* transfection capacity in different fish tissues as detected by fluorescence. The expression of 13 immune-related genes was increased, and expressions were maintained until day 70 in the spleen and hindgut of vaccinated fish. The survival percentages of immunized fish were 63.33%, 85.33%, and 59.33% when the challenges were performed at 3, 21, and 70 dpi, respectively. This strategy demonstrates how NPs can generate robust immune responses in fish and activate antigen-presenting cells. A strategy included the G131c antigen sequence in the CH3 region of carp IgM [35], and the construct was packaged with chitosan and PLGA. The idea was that transfected cells from immunized fish would express the fused protein, and antigen-presenting cells would recognize it and process the antigen, inducing a protective immune response. NPs were prepared using the double-emulsion methodology, generating NPs of 237.42–287.62 nm, and fish were immunized with 2 µg/g (body weight). After that, challenges were made at 28 and 70 dpv. NPs released DNA from 6 to 168 h, without affecting cell viability. The transfection of spleen cells from immunized fish was also evaluated, as was the expression of several genes related to innate and adaptive immunity, revealing overexpression of IgM at 28 dpv. Vaccination-induced antibody titers and neutralizing antibodies were higher at 28 dpv. Finally, survival rates after the challenge were 83.78% and 72.97% at 28 dpv and 70 dpv, respectively. This study showed that this antigen presentation strategy, combined with NPs, can aid vaccine development and promote effective vaccination.

For this disease, NPs were administered intraperitoneally to carp or zebrafish, which is not an easy way to perform large-scale vaccination, and the *G* gene was used as the target. However, the dose was constant in studies (2 µg/g), and the protection rate was not similar, reaching protection rates of 85.33% after 21 days post-vaccination (dpv) in zebra fish or 83.78% in carp, but after 70 dpv, protection was 59.33% and 72.97% in zebra fish and carp, respectively.

### White Spot Syndrome Virus

The White spot syndrome virus (WSSV) is an enveloped DNA virus that affects shrimp, causing up to 100% mortality. It is distributed worldwide, and there is no form of infection prevention. There is a great need to develop a vaccine; however, shrimp lack an adaptive immune system and have only an innate immune system. However, some studies have reported that the use of inactivated agents can help prevent infection [36].

Rajeshkumar *et al*. [37] evaluated the use of a gene vaccine encoding the *VP28* gene of WSSV, which was encapsulated in chitosan. NPs were included in the shrimp feed so that it could be administered to many shrimps. Feed (10 g) containing 200 ml of chitosan and 1 ml of DNA. This study showed that VP28 expression was detected in the transfected sea bass kidney cell line. Shrimp fed NPs also showed increased protein expression in different tissues (gut, gills, pleopods, head soft tissue, and hepatopancreas). Shrimp were challenged, and they showed survival rates of 90%, 76.66%, and 56.66% at 7, 14, and 30 days postoperatively, respectively. The immunological parameters of the shrimp (prophenoloxidase activity, superoxide dismutase, and superoxide anion levels) were measured. Parameters in orally vaccinated shrimp were higher, indicating a protective effect and immune response activation.

In another study, NPs containing the *VP28* gene were prepared by ionic gelation using chitosan and tripolyphosphate (TPP). They were mixed with feed (10 g) containing 3 ml chitosan/TPP and 100 µg/ml of the shrimp construct [38]. The toxicity of NPs in cells was evaluated at different chitosan concentrations, and no toxicity was observed, with the lowest survival rate above 85% at 5 mg of NPs. The expression of VP28 was detected in different tissues of immunized shrimp by dot-blot analysis. This strategy demonstrated that oral vaccination in shrimp using (CN)/TPP.

For WSSV, studies have reported oral NPs delivery via food, allowing massive vaccination using *VP28* as a target gene; however, only one study reported a protection rate that decreased over time. However, it is important to highlight that shrimp have a different immune system than mammals, which is why the expression of prophenoloxidase and superoxide dismutase was reported as an immune response activity.

### Miscellaneous Viruses

The poultry industry requires the development of vaccines because it is affected by several pathogens, which can impair chicken development and, consequently, feed production. The avian reticuloendothelial hyperplasia virus causes tumor formation and dwarfism. Infected birds are susceptible to opportunistic infections due to immunosuppression.

*gp90* is a vaccine candidate gene, and in one study, it was packaged with chitosan and the NPs were coated with erythrocyte membranes [39]. One-day-old chickens were immunized with 1 µg/g body weight IM and received a booster 14 days later. The immunized birds showed increased NO synthesis and iNOS expression, which are macrophage effector molecules in inflammatory responses. They also showed increases in IL-4 and IFN-γ production and in the CD4+ lymphocyte population. The expression of several genes regulating the immune response (IFIHI, TLR3, and viperin) was also increased, and protection against the challenge was observed at 100% with 30 dpv, whereas it was not achieved with 3 dpv due to possible immunosuppression caused by the virus.

Zhang *et al*. [40] evaluated NPs against the avian influenza virus H9N2 [40]. In this study, chickens were immunized three times. The first two were immunized with a DNA vaccine harbored in *Salmonella* as a carrier, and the third immunization was performed in an NP format. *M1* and *HA* genes were used as target genes that caused IgG and sIgA production in chickens. It was reported that CD8+ cells from immunized chickens proliferated significantly more than controls, whereas no significant difference was observed in CD4+ cells. In terms of cytokine expression, IFN-gamma expression, which is produced by Th1 cells, was higher than that in controls. After the challenge, all chickens survived, and protection was reported by weight growth rate, with vaccinated chickens showing the highest rate. Virus shedding was measured by qPCR, and vaccinated chickens showed the lowest level of oropharyngeal virus shedding.

The influenza virus is spreading worldwide, and the development of an effective vaccine is an aim of many laboratories. In this work, the authors showed the impact of a DNA vaccine harbored in bacteria, which can work as an adjuvant. This strategy is called a protein/DNA boost. However, there are some questions about the dose because it is not described how much plasmid was used on any application day. Whether the elicited immune response was caused by bacterial pathogen-associated molecular patterns, the *GM-CSF* gene, or the target genes remains unclear.

Infectious hematopoietic necrosis virus causes mortality in various fish species, including salmon, brook trout, and rainbow trout. A *VP2* gene vaccine was constructed using chitosan in one study. NPs were prepared using TPP or alginate [41] and administered orally via ionic gelation. Immunization and boost with 10 and 25 µg of DNA were performed, and after 30 dpv, VP2 expression was detected in various tissues of immunized fish, indicating *in vivo* transfection. Subsequently, the expression of IFN-1 and MX-1, as well as the *IgM* and *IgT* genes, was increased in the NP-vaccinated groups. CD4 gene expression was also increased. Then, survival rates were 76% and 90% for fish vaccinated with alginate with 10 µg or 25 µg, respectively, while CN -vaccinated fish reached 70% and 83% for 10 µg and 25 µg, respectively. Interestingly, VP4 expression was evaluated in vaccinated and challenged fish and researchers detected that expression was decreased in the spleen and liver, indirectly indicating that viral replication was lower.

Grass carp hemorrhagic virus affects fish in Asia. In one study, nanoparticle formation was performed using the ionic gelation method. The plasmid contained the viral genes *VP4C* and *VP56C* [42]. NPs were administered orally, and carps were challenged 10 days after the last immunization. One remarkable aspect was that expressions of inflammation-related genes (IL-1β and TNFα) were upregulated, and expression decreased over time. In addition, virus replication was decreased in all vaccinated groups, demonstrating that an oral NP vaccine can induce systemic protection and has the potential to be used in mass vaccination strategies.

In this part, the avian reticuloendotheliosis NP vaccine was delivered twice in 2 weeks [39]; however, the DNA–NP dose (1 µg/g of body weight) was lower than in some other studies in chickens and with other viruses because 100 or 200 µg of genetic material was used. The reported protection rate increased by 100%, and the delivery route was IM.

Infectious pancreatic necrosis virus and grass carp hemorrhagic disease reports used an oral delivery without and with flagellin as an adjuvant, respectively. Oral delivery for both diseases allows massive distribution in fish factories. One important aspect is that, in a bank fish, the amount of vaccine each individual consumes is uncertain, allowing some to consume large amounts and others none.

## CONCLUSION

Vaccine development continues to expand rapidly as the need to maintain healthy animal populations grows. Chitosan-based DNA nanoparticles present several advantages as vaccine platforms: they enable tissue- and cell-specific targeting, protect genetic material in the extracellular environment, and provide controlled release. These nanoparticles enhance transfection efficiency compared with naked DNA, improve safety, and exhibit low toxicity. Unlike killed or attenuated vaccines, DNA–NP vaccines do not require the cultivation or replication of pathogens, thereby reducing biosafety risks and minimizing the possibility of vaccine virus spillover. Moreover, DNA–NP vaccines can function as Differentiating Infected from Vaccinated Animals (DIVA) tools, supporting the identification of circulating viral strains and reducing opportunities for viral recombination. Their flexibility also permits multiple booster doses, incorporation of several antigenic targets, and integration of various genetic adjuvants, enabling the formulation of multicomponent vaccines capable of eliciting targeted humoral or cellular responses.

The versatility of chitosan nanoparticles has been demonstrated across species, inducing systemic and mucosal immunity in mammals and poultry and stimulating protective innate mechanisms in species lacking adaptive immunity, such as shrimp. Although several nanoparticle formulation methods exist, ionic gelation and complex coacervation remain the most common. These techniques, however, require further optimization, as nanoparticle characteristics depend on plasmid size, gene sequence, and required dose. Most published studies involve guinea pigs, rodents, poultry, or aquatic animals; consequently, dose optimization and large-scale formulation protocols for larger species remain insufficiently explored. In addition, nanoparticle stability during storage is a critical knowledge gap. Nearly all current studies utilize freshly prepared nanoparticles, and the long-term stability, immunogenicity, and protective efficacy of vaccine NPs stored for 3 to 6 months remain unknown.

Regulatory limitations also affect the application of chitosan in veterinary vaccines. Although chitosan is classified as GRAS by the FDA and approved for use in foods, agencies such as the United States Department of Agriculture have not yet approved its use as a veterinary vaccine component, restricting it primarily to pollution control technologies [43]. Nevertheless, the ability to administer nanoparticles via oral and nasal routes, both highly desirable for mass vaccination, makes them particularly suitable for poultry and aquaculture industries. Notably, many studies achieving 100% protection utilized nanoparticles smaller than ~300 nm, underscoring the importance of particle size. Still, large-scale production strategies and stability studies remain lacking.

Beyond vaccines, chitosan nanoparticles hold broader potential as carriers for drugs, adjuvants, RNA, iRNA, linear DNA, and bioactive molecules. Their mucoadhesive properties extend residence time at mucosal surfaces, enhancing local delivery and therapeutic efficacy [44]. Nanoparticles can also be conjugated with antibodies, peptides, or scFv fragments to improve cell-specific targeting, reduce required doses, and enhance delivery efficiency. Their demonstrated effectiveness across species with adaptive immune systems (e.g., chickens, fish) and those relying solely on innate immunity (e.g., shrimp) highlights their significant versatility and potential for rapid, low-cost, and even personalized vaccine development.

Overall, chitosan-based DNA nanoparticles represent a promising platform for developing vaccines against viral diseases in both small and large animals. However, future research must address critical gaps, including large-animal studies, field evaluations against wild-type viruses, scalability, regulatory approval pathways, and NPs stability, to fully realize their potential in modern veterinary immunization.

## AUTHORS’ CONTRIBUTIONS

JAC-B: Conceptualization, MG-L and JAC-B: Investigation and drafted and edited the manuscript. Both authors have read and approved the published version of the manuscript.
